# A dataset for ^15^N natural abundance of soil ammonia volatilization

**DOI:** 10.1038/s41597-024-04216-w

**Published:** 2024-12-18

**Authors:** Lingyun Peng, Chaopu Ti, Bin Yin, Xiao Bai, Miao Li, Limin Tao, Xiaoyuan Yan

**Affiliations:** 1 State Key Laboratory of Soil and Sustainable Agriculture, Changshu National Agro-Ecosystem Observation and Research Station, Institute of Soil Science, Chinese Academy of Sciences, Nanjing, 210008 China; 2 University of Chinese Academy of Sciences, Beijing, 100049 China

**Keywords:** Environmental impact, Agroecology, Element cycles

## Abstract

Investigating the sources of ammonia (NH_3_) in the atmosphere and the contribution of each source is essential for environmental pollution control. The presented dataset aims to provide ^15^N natural abundance (δ^15^N) data collected from different controlled treatments to fill the knowledge gap between insufficient data of soil δ^15^N-NH_3_ and accurately identifying atmospheric NH_3_ source apportionments. Our results showed that the overall δ^15^N-NH_3_ values emitted from soil ranged from −46.09 to 10.22‰, with an average of −26.81 ± 11.17‰. The mean δ^15^N-NH_3_ values under different nitrogen (N) application rates, N fertilizer types, air temperatures, soil moisture, soil pH, soil types, and land use types were −29.41 ± 10.91, −32.43 ± 6.86, −29.10 ± 10.04, −30.31 ± 6.18, −24.84 ± 13.76, −23.53 ± 7.66, and −14.57 ± 12.54‰, respectively. Significant correlations were observed between δ^15^N-NH_3_ values and soil pH, soil NO_3_^−^-N concentration, and NH_3_ volatilization. This unique database provides basic data and evidence for the qualification of atmospheric NH_3_ source apportionments under different study area conditions.

## Background & Summary

Food security and air quality are two major issues that currently exist and will persist for a long time, with complex causal relationships that need to be balanced^1–3^. Excessive nitrogen (N) input increased crop yields while resulted in significant losses of reactive N, particularly ammonia (NH_3_) emissions, which have gradually become a constraining factor affecting the balance^4–6^. Approximately 40% of total NH_3_ is emitted from N fertilizer application, which has resulted in air pollution, soil acidification, water eutrophication, and biodiversity loss^7–11^. Therefore, identifying and quantifying the contribution of N fertilizer application to atmospheric NH_3_ is vital in better understanding the pathways for NH_3_ emission reduction and determining mitigation options.

The use of ^15^N natural abundance (δ^15^N) technology is a complementary tool to identify and quantify the sources of atmospheric NH_3_ owing to its relatively distinct and well-defined N isotopic signature^12–16^. Previous studies have found that the δ^15^N values of NH_3_ emitted from agricultural sources, such as volatilized fertilizer (−46 ± 5‰) and livestock (−28 ± 11‰), were considerably lower than those emitted from fossil fuel sources (−8 ± 6‰), marine emissions (−10 ± 3‰) and other sources, as reported by Elliott, *et al*.^17^ and Bhattarai, *et al*.^18^. Therefore, according to the distinct signature of δ^15^N-NH_3_ values, the contribution of soil NH_3_ emissions to atmospheric NH_3_ can successfully be quantified at present^18–20^. For instance, through the observation of δ^15^N-NH_3_ values from major local emissions, the combined contribution of cropland and livestock was found to account for 64.5% of the total NH_3_ emissions in the Beijing-Tianjin-Hebei region^20^.

Some studies have investigated δ^15^N-NH_3_ values emitted from agricultural sources, but most of them used fixed isotope values to quantify the contribution of cropland volatilization to atmospheric NH_3_. However, source identification requires knowledge of study area conditions and source emission characteristics. NH_3_ volatilization is influenced by various factors such as fertilizer application rate, soil NH_4_^+^ concentration, and temperature^21–24^. These factors directly or indirectly lead to large changes in the δ^15^N value of NH_3_ volatilization^25–27^. For instance, temperature is negatively correlated with the δ^15^N-NH_3_ value according to field observations^19^. Moreover, Cejudo and Schiff^28^ stated that the higher the water pH, the easier the volatilization of ^14^N, resulting in a lower volatilized δ^15^N value. Many factors influence the δ^15^N-NH_3_ value in the volatilization process of soil NH_3,_ and certain changes can be expected in the temporal or spatial scales. Therefore, the characteristics of δ^15^N-NH_3_ values must be studied under different conditions as the use of fixed coefficients may affect the accuracy of traceability results^29^.

Owing to the complexity of the influencing factors, there is an urgent need to precisely analyze the influence of various factors on δ^15^N-NH_3_ values to provide a research basis for subsequent traceability studies. To fill this knowledge gap, we established a comprehensive database on δ^15^N-NH_3_ values from soils controlled by seven factors. This database is available from the Figshare repository. The results in this database provided basic data of δ^15^N-NH_3_ values for the entire volatilization process in soils and improved the accuracy of the traceability analysis of atmospheric NH_3_.

## Methods

### Experiment design

To investigate the δ^15^N values of soil NH_3_ volatilization under different conditions, seven controlled laboratory incubation experiments were conducted using the sponge-trapping method. The experiments included fertilization factors (N application rate, and N fertilizer type), meteorological factors (air temperature), soil factors (soil moisture, soil pH, and soil type), and land use type. The detailed experimental design is presented in Table 1.

**Table 1:** Incubation conditions for the seven experiments (we used sulfuric acid and sodium hydroxide for soil conditioning at different pH levels (pH5, pH6, pH7, and pH8) in Exp5 using soils from Changshu).

Experiment	Control factor	Treatment	Air humidity	Temperature	N application rate	Soil moisture
Exp1	N application rate	0/20/180/360 kg N ha^−1^	95 ± 5%	25 °C	—	WFPS60%
Exp2	N fertilizer type	Urea/Urease inhibitor/Compound fertilizer/Ammonium nitrate phosphate fertilizer	95 ± 5%	25 °C	180 kg N ha^−1^	WFPS60%
Exp3	Air temperature	5/15/25/35 °C	95 ± 5%	—	180 kg N ha^−1^	WFPS60%
Exp4	Soil moisture	WFPS 40/60/80/100%	95 ± 5%	25 °C	180 kg N ha^−1^	WFPS60%
Exp5	Soil pH	pH 5/6/7/8	95 ± 5%	25 °C	180 kg N ha^−1^	WFPS60%
Exp6	Soil type	Northeast (Cinnamon soil)/ Central (Fluvo-aquic soil)/ North (Meadow cinnamon)/ Southwest (Brown soil) China	95 ± 5%	25 °C	180 kg N ha^−1^	SWC60%
Exp7	Land use type	Vineyard/Vegetable /Forest	95 ± 5%	25 °C	180 kg N ha^−1^	WFPS60%

Two forms of soil moisture were addressed in the table, including water-filled pore space (WFPS) and soil water content (SWC). The N fertilizer types of Exp1, and Exp 3–7 were urea.

### Experimental materials

Soil samples were collected from the surface layer of the soil (0–20 cm). Soils of Exp1, 2, and 4 were collected in mid-November 2018 and those of Exp 3 and 5 were collected in mid-November 2019 from Changshu Agro-ecological Experimental Station (31° 32′ 93″ N, 120° 41′ 88″ E), Jiangsu province in eastern China. Soils of Exp6 were collected with the same topography, fertilization, tillage practices, and crop growth conditions, from early September to mid-October 2020 from Beipiao, Liaoning province in northeastern China (41° 57′ N, 120° 36′ E), Xinxiang, Henan province in central China (35° 60′ N, 113° 56′ E), Tangshan, Hebei province in northern China (39° 47′ N, 118° 0′ E), and Linzhi, Tibet in the highlands of southwestern China (29° 34′ N, 94° 25′ E), respectively. Soils of the bamboo forest in Exp7 were collected at Nanjing, Jiangsu province (31° 16′ N, 118° 53′ E), whereas those of vineyard and vegetable growth were collected from Changshu Agro-ecological Experimental Station, Jiangsu province (31° 32′ 93″ N, 120° 41′ 88″ E). Roots and visible rocks in the soil were carefully removed manually, and the soil sample was then air-dried and ground to pass through a 2-mm stainless steel sieve to achieve a high degree of homogeneity. The physicochemical properties of the different sets of soils are listed in Table 2. The δ^15^N values of urea, compound fertilizer, and ammonium nitrate phosphate fertilizer were −3.6 ± 0.1, −3.0 ± 0.4, and −0.8 ± 0.7‰, respectively.

**Table 2:** Basic properties of different sets of soil.

Sets	Land use types	Soil types	Area	pH	TN (g/kg)	NH_4_^+^-N (mg/kg)	NO_3_^−^-N (mg/kg)	Clay (%)	Silt (%)	Sand (%)
Exp1,2,4	Cropland	Gleyi-Stagnic Anthrosol	Changshu	7.09	0.27	3.55	6.10	31.70	58.40	9.90
Exp3,5	Cropland	Gleyi-Stagnic Anthrosol	Changshu	7.30	0.31	7.33	3.82	31.50	57.90	10.60
Exp 6	Cropland	Cinnamon soil	Beipiao	6.63	0.75	5.63	4.21	19.80	16.70	63.50
Exp 6	Cropland	Fluvo-aquic soil	Xinxiang	8.07	0.82	4.23	85.97	18.50	22.90	58.60
Exp 6	Cropland	Meadow cinnamon soil	Tangshan	6.44	0.91	7.41	29.67	23.57	21.63	54.80
Exp 6	Cropland	Brown soil	Linzhi	5.83	0.95	0.74	57.04	8.66	17.94	73.40
Exp 7	Vineyard	Gleyi-Stagnic Anthrosol	Changshu	6.37	0.20	6.69	45.30	31.04	58.06	10.90
Exp 7	Vegetable	Gleyi-Stagnic Anthrosol	Changshu	6.99	0.19	3.66	316.00	36.74	56.02	7.58
Exp 7	Forest	Yellow-brown soil	Nanjing	5.52	0.16	5.18	4.17	38.14	49.34	13.70

### Soil physical and chemical analysis

The concentrations of soil NH_4_^+^-N and NO_3_^−^-N were determined using a continuous-flow analyzer (Skalar San^++^ System, Breda, Netherlands) after filtering, the addition of 5 g of post-culture soil to 50 mL of 2 mol L^−1^ KCl solution and shaking for 1 h. The minimum detection limits for the NH_4_^+^-N and NO_3_^−^-N concentrations were 0.046 and 0.015 mg N L^−1^, respectively. Subsequently, soil was removed from the culture bottles to dry in natural air. The soil texture was assessed using the laser diffraction method, soil pH was measured using a glass electrode in a soil: water suspension at 1:2.5(v/v), and soil total nitrogen (TN) content was determined using a Vario Max CN analyzer (Elementar, Vario Max CN, Hanau, Germany) through the dry burning method.

### NH_3_ volatilization measurements

**Fig. 1:**
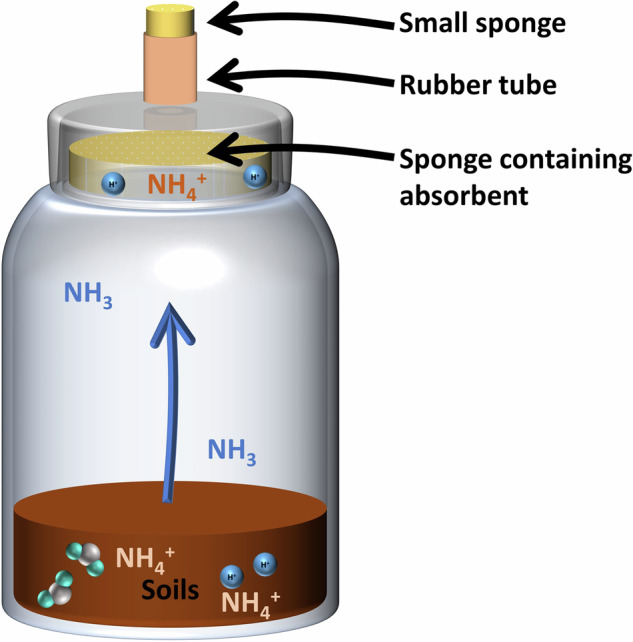
The schematic diagram of incubation.

In this study, the sponge-trapping method described by Ti, *et al*.^30^ was employed to measure NH_3_ volatilization under different influencing factors in controlled laboratory incubation experiments. In particular, the NH_3_ emitted from soil was absorbed in a sponge with an acid solution for staged cultivation. To capture NH_3_ emissions from the soil, a sponge with a diameter and thickness of 8.5 and 1 cm, respectively, containing 4 mL of glycerol phosphate absorbent was positioned on the neck of a 500-mL incubation bottle. The bottle cap featured a hole with a diameter of 1.4 cm, into which a rubber tube with a diameter of 1.2 cm was inserted. A small sponge with absorbent was placed in this hole to prevent the loss of NH_3_ from the bottle into the air (Fig. 1).

The experiment, designed for non-destructive sampling with three replicates, ensured data accuracy. Dry-weight soil was placed in an incubation bottle, and soil moisture was adjusted to a specific percentage of WFPS or SWC by adding deionized water. The specific incubation conditions for the seven sets of experiments are listed in Table 2. Trapping sponges and incubated soils were sampled on days 1, 2, 3, 4, 5, 6, 7, and 15 (excluding day 7 for experiment Exp6). At each sampling interval, the removed trapping sponges were plunged into 50 mL of 1 mol L^−1^ KCl, shaken for 2.5 h at 100 rotations per minute for NH_4_^+^-N sample extraction, and analyzed for NH_4_^+^-N using a continuous-flow analyzer.

### N isotopic analysis

The δ^15^N-NH_3_ values were measured using the method described by Liu, *et al*.^31^. This method relies on the isotopic analysis of nitrous oxide (N_2_O). By examining the linear correlation between the δ^15^N values of the substrate (NH_4_^+^) and resulting gas (N_2_O), a standard curve was fitted, enabling the deduction of the δ^15^N-NH_4_^+^ values of the substrate. The isotope ratio is reported in parts per thousand relative to atmospheric N_2_ according to Eq. (1).

1 $${\delta }^{15}{{\rm{N}} \mbox{-} {\rm{NH}}}_{{\rm{x}}}(\textperthousand )=\frac{{({\rm{N}}15/{\rm{N}}14)}_{{\rm{sample}}-}{({\rm{N}}15/{\rm{N}}14)}_{{\rm{standard}}}}{{({\rm{N}}15/{\rm{N}}14)}_{{\rm{standard}}}}\times 1000$$

The N isotopic compositions of all the samples were analyzed using an isotope mass spectrometer (Isoprime 100, Isoprime, UK). International reference δ^15^N-NH_4_^+^ standards, namely, USGS25 (−30.4‰), USGS26 (+53.7‰), and IAEAN1 (+0.4‰), were chosen for data correction purposes.

## Data Records

The dataset is available at Figshare^32^, an open-access repository where users can make all their research outputs available in a citable, shareable, and discoverable manner. The Excel file was named Natural Isotopic Abundance of Soil Ammonia Volatilization Excel Data.xlsx. This file provided complete information on the seven controlled experiments, including experiment sets (array named Set), days of incubation (array named Day), experimental treatment (array named Treatment), the mean values of δ^15^N-NH_3_, cumulative NH_3_, soil NH_4_^+^-N, soil NO_3_^−^-N, soil pH, and their standard deviations (array named sd). The data for Exp1, Exp2, Exp6, and Exp7 have been published and were described in detail^29,30,33,34^_._

## Technical Validation

The data were collected and measured using a standardized protocol and calibrated continuous-flow analyzer and an isotope mass spectrometer. Each experiment was repeated thrice to ensure the accuracy of the results. The data were analyzed using appropriate statistical methods to identify the significant effects of the different factors on δ^15^N-NH_3_ values emitted from soils.

## Usage Notes

This dataset provides a valuable resource for researchers and policymakers, as well as those who are interested in agricultural sources of atmospheric NH_3_ and effective strategies to reduce NH_3_ emissions and protect the environment. This dataset can also be used to validate the “bottom-up” emission inventory methodology and model simulations.

## Data Availability

No custom code was used for the curation and/or validation of the dataset.
